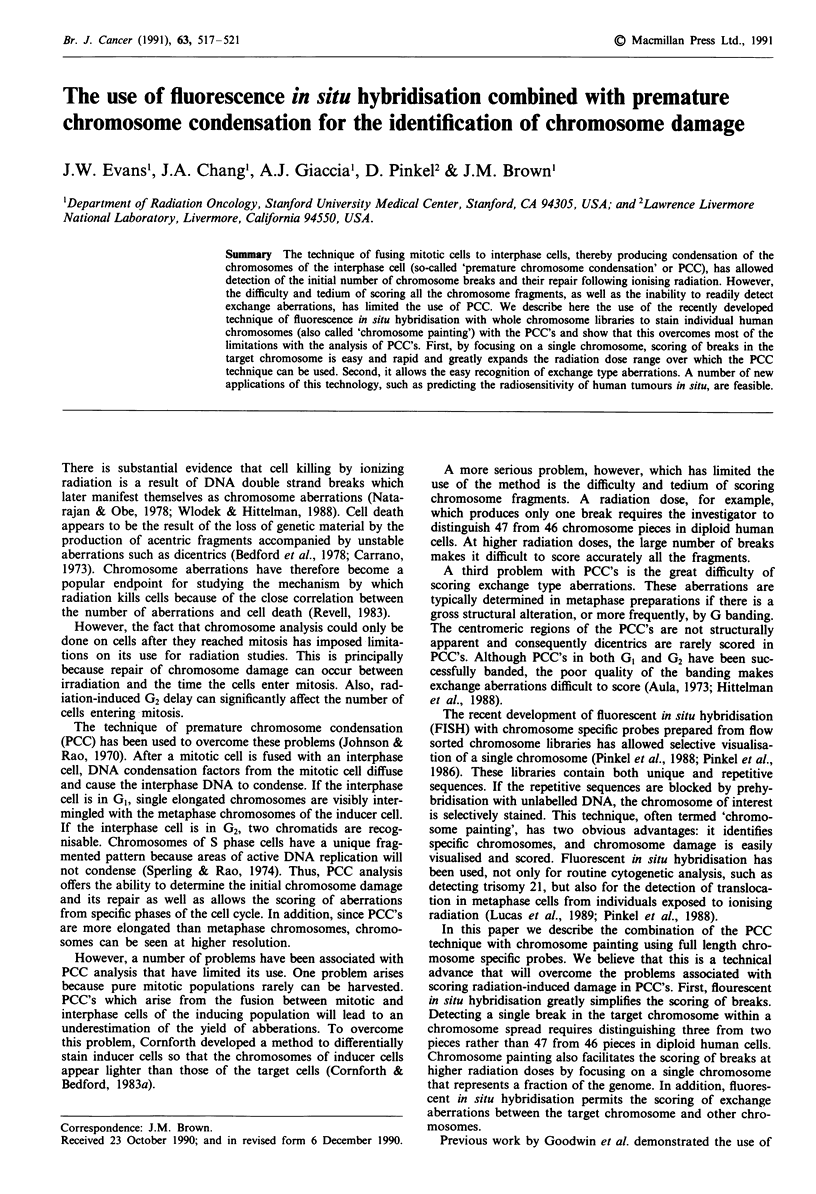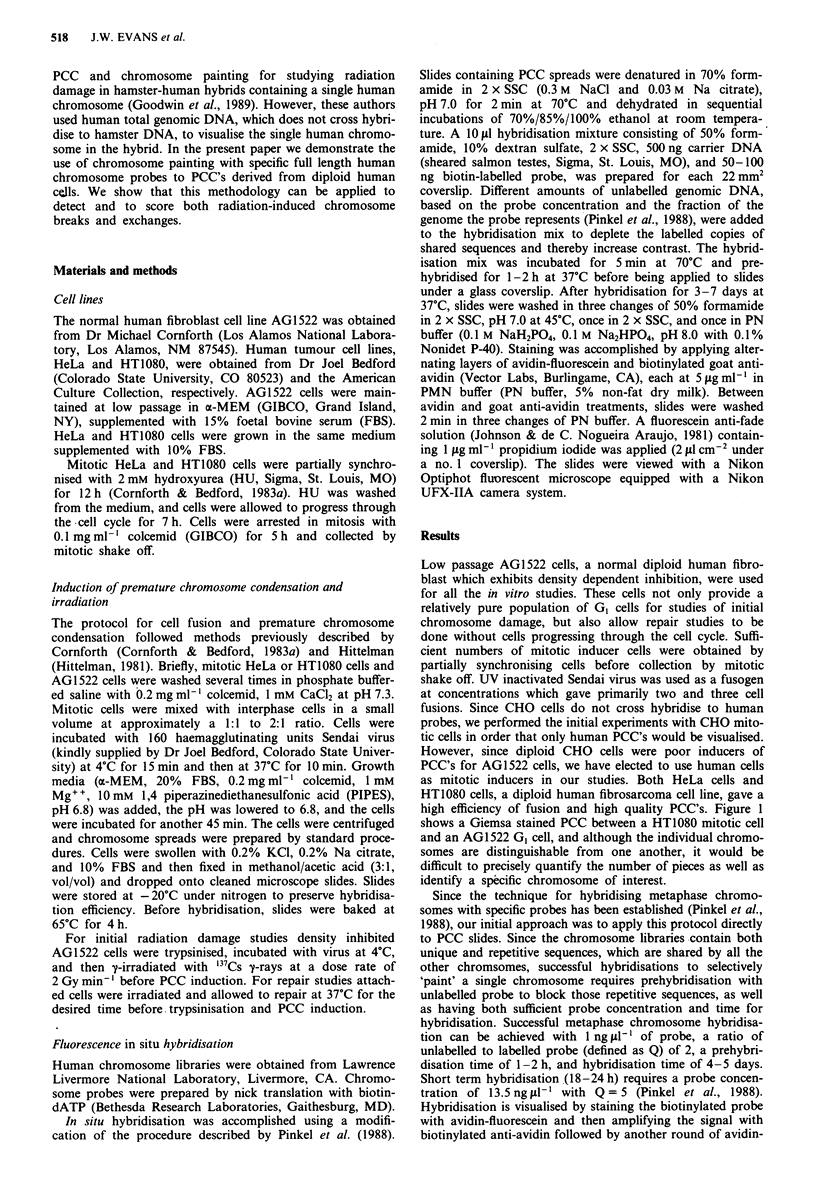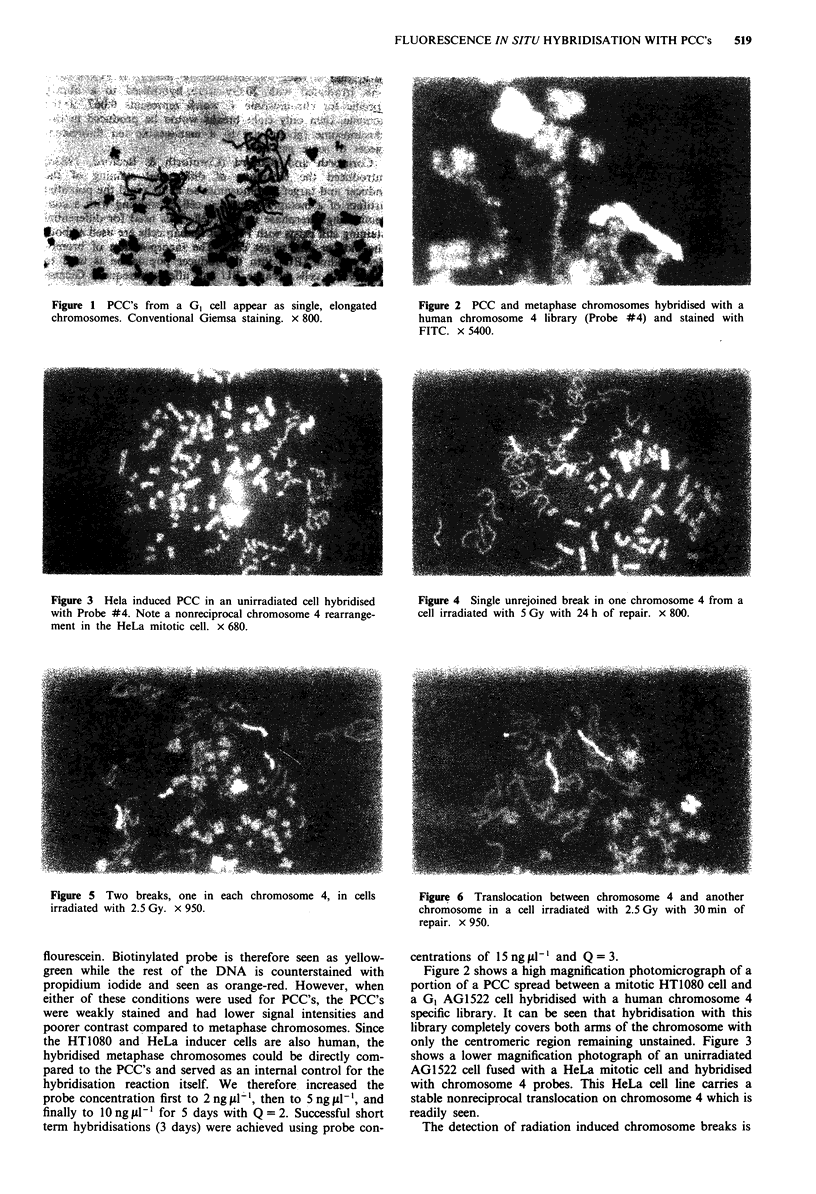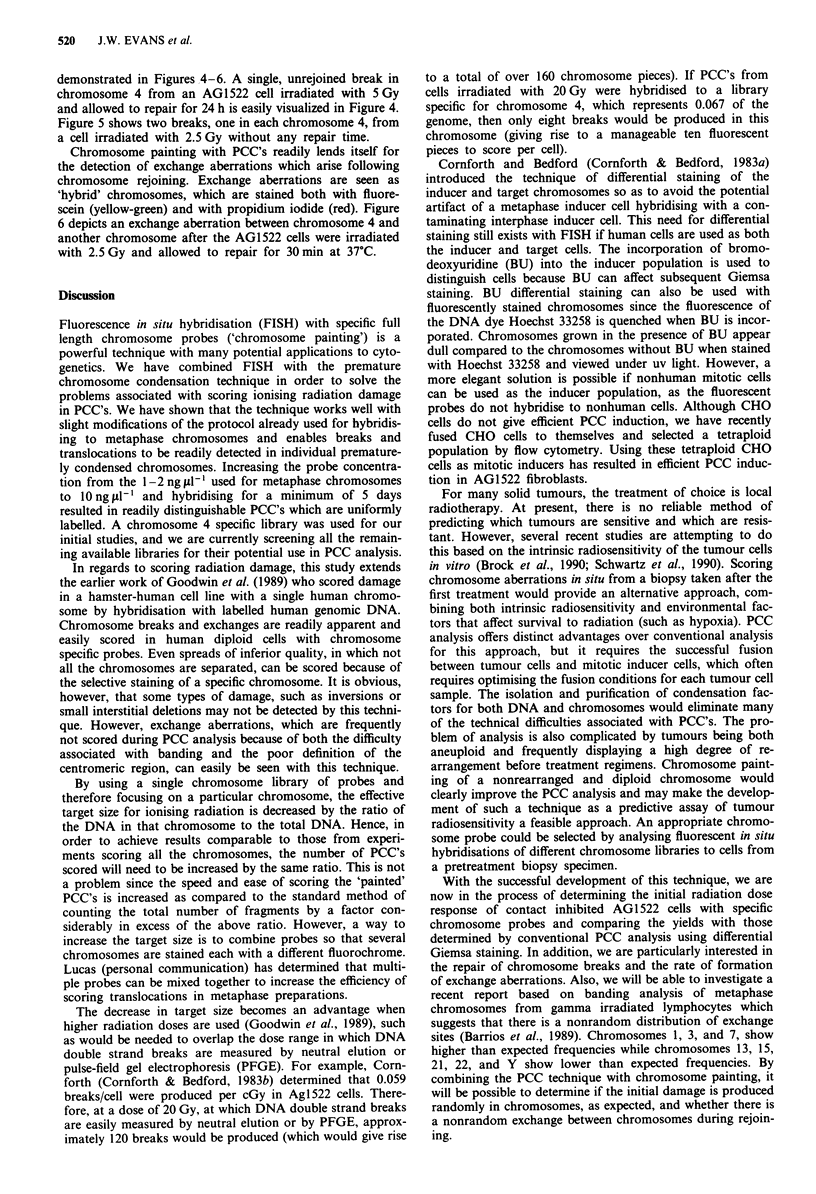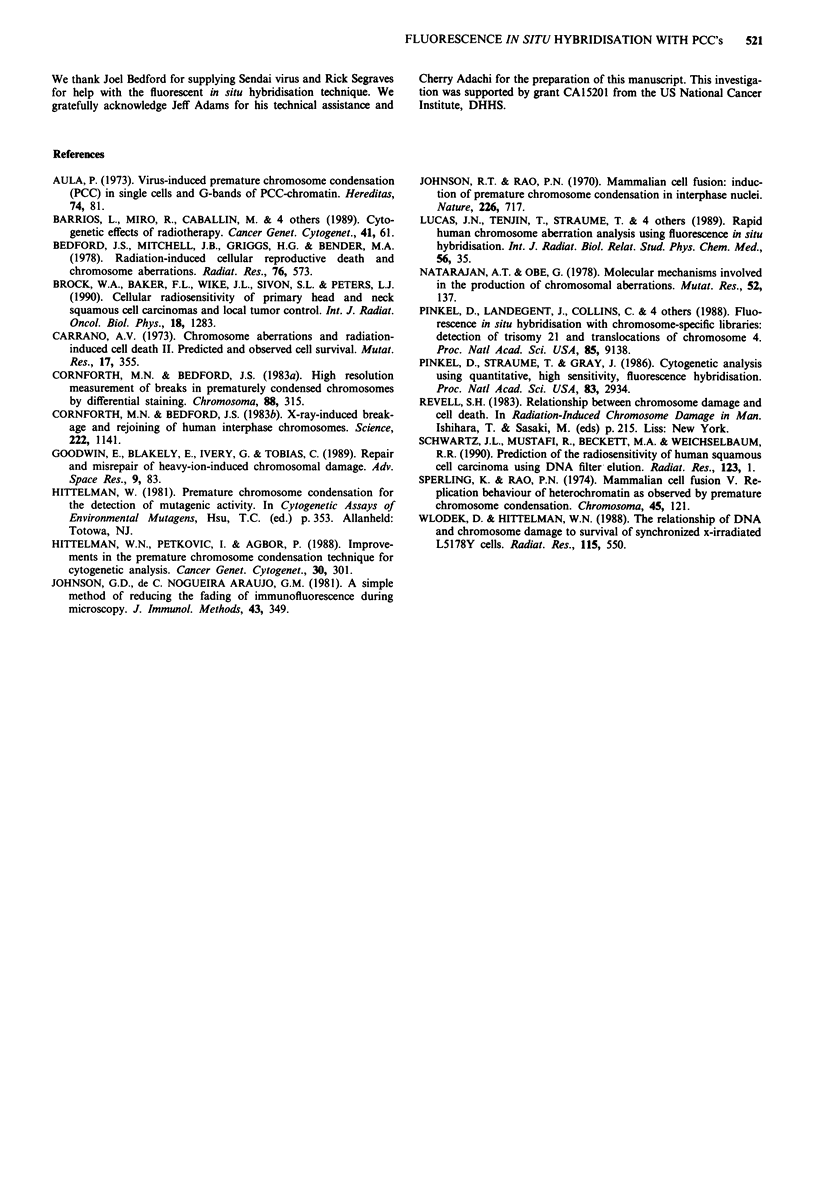# The use of fluorescence in situ hybridisation combined with premature chromosome condensation for the identification of chromosome damage.

**DOI:** 10.1038/bjc.1991.123

**Published:** 1991-04

**Authors:** J. W. Evans, J. A. Chang, A. J. Giaccia, D. Pinkel, J. M. Brown

**Affiliations:** Department of Radiation Oncology, Stanford University Medical Center, CA 94305.

## Abstract

**Images:**


Br. J. Cancer (1991), 63, 517–521
© Macmillan Press Ltd., 1991

### The use of fluorescence *in situ* hybridisation combined with premature chromosome condensation for the identification of chromosome damage

J.W. Evans[1], J.A. Chang[1], A.J. Giaccia[1], D. Pinkel[2] & J.M. Brown[1]

[1]*Department of Radiation Oncology, Stanford University Medical Center, Stanford, CA 94305, USA; and* [2]*Lawrence Livermore National Laboratory, Livermore, California 94550, USA.*

**Summary** The technique of fusing mitotic cells to interphase cells, thereby producing condensation of the chromosomes of the interphase cell (so-called 'premature chromosome condensation' or PCC), has allowed detection of the initial number of chromosome breaks and their repair following ionising radiation. However, the difficulty and tedium of scoring all the chromosome fragments, as well as the inability to readily detect exchange aberrations, has limited the use of PCC. We describe here the use of the recently developed technique of fluorescence *in situ* hybridisation with whole chromosome libraries to stain individual human chromosomes (also called 'chromosome painting') with the PCC's and show that this overcomes most of the limitations with the analysis of PCC's. First, by focusing on a single chromosome, scoring of breaks in the target chromosome is easy and rapid and greatly expands the radiation dose range over which the PCC technique can be used. Second, it allows the easy recognition of exchange type aberrations. A number of new applications of this technology, such as predicting the radiosensitivity of human tumours *in situ*, are feasible.

There is substantial evidence that cell killing by ionizing radiation is a result of DNA double strand breaks which later manifest themselves as chromosome aberrations (Natarajan & Obe, 1978; Wlodek & Hittelman, 1988). Cell death appears to be the result of the loss of genetic material by the production of acentric fragments accompanied by unstable aberrations such as dicentrics (Bedford *et al*., 1978; Carrano, 1973). Chromosome aberrations have therefore become a popular endpoint for studying the mechanism by which radiation kills cells because of the close correlation between the number of aberrations and cell death (Revell, 1983).

However, the fact that chromosome analysis could only be done on cells after they reached mitosis has imposed limitations on its use for radiation studies. This is principally because repair of chromosome damage can occur between irradiation and the time the cells enter mitosis. Also, radiation-induced $G_2$ delay can significantly affect the number of cells entering mitosis.

The technique of premature chromosome condensation (PCC) has been used to overcome these problems (Johnson & Rao, 1970). After a mitotic cell is fused with an interphase cell, DNA condensation factors from the mitotic cell diffuse and cause the interphase DNA to condense. If the interphase cell is in $G_1$, single elongated chromosomes are visibly intermingled with the metaphase chromosomes of the inducer cell. If the interphase cell is in $G_2$, two chromatids are recognisable. Chromosomes of S phase cells have a unique fragmented pattern because areas of active DNA replication will not condense (Sperling & Rao, 1974). Thus, PCC analysis offers the ability to determine the initial chromosome damage and its repair as well as allows the scoring of aberrations from specific phases of the cell cycle. In addition, since PCC's are more elongated than metaphase chromosomes, chromosomes can be seen at higher resolution.

However, a number of problems have been associated with PCC analysis that have limited its use. One problem arises because pure mitotic populations rarely can be harvested. PCC's which arise from the fusion between mitotic and interphase cells of the inducing population will lead to an underestimation of the yield of abberations. To overcome this problem, Cornforth developed a method to differentially stain inducer cells so that the chromosomes of inducer cells appear lighter than those of the target cells (Cornforth & Bedford, 1983*a*).

Correspondence: J.M. Brown.
Received 23 October 1990; and in revised form 6 December 1990.

A more serious problem, however, which has limited the use of the method is the difficulty and tedium of scoring chromosome fragments. A radiation dose, for example, which produces only one break requires the investigator to distinguish 47 from 46 chromosome pieces in diploid human cells. At higher radiation doses, the large number of breaks makes it difficult to score accurately all the fragments.

A third problem with PCC's is the great difficulty of scoring exchange type aberrations. These aberrations are typically determined in metaphase preparations if there is a gross structural alteration, or more frequently, by G banding. The centromeric regions of the PCC's are not structurally apparent and consequently dicentrics are rarely scored in PCC's. Although PCC's in both $G_1$ and $G_2$ have been successfully banded, the poor quality of the banding makes exchange aberrations difficult to score (Aula, 1973; Hittelman *et al*., 1988).

The recent development of fluorescent *in situ* hybridisation (FISH) with chromosome specific probes prepared from flow sorted chromosome libraries has allowed selective visualisation of a single chromosome (Pinkel *et al*., 1988; Pinkel *et al*., 1986). These libraries contain both unique and repetitive sequences. If the repetitive sequences are blocked by prehybridisation with unlabelled DNA, the chromosome of interest is selectively stained. This technique, often termed 'chromosome painting', has two obvious advantages: it identifies specific chromosomes, and chromosome damage is easily visualised and scored. Fluorescent *in situ* hybridisation has been used, not only for routine cytogenetic analysis, such as detecting trisomy 21, but also for the detection of translocation in metaphase cells from individuals exposed to ionising radiation (Lucas *et al*., 1989; Pinkel *et al*., 1988).

In this paper we describe the combination of the PCC technique with chromosome painting using full length chromosome specific probes. We believe that this is a technical advance that will overcome the problems associated with scoring radiation-induced damage in PCC's. First, flourescent *in situ* hybridisation greatly simplifies the scoring of breaks. Detecting a single break in the target chromosome within a chromosome spread requires distinguishing three from two pieces rather than 47 from 46 pieces in diploid human cells. Chromosome painting also facilitates the scoring of breaks at higher radiation doses by focusing on a single chromosome that represents a fraction of the genome. In addition, fluorescent *in situ* hybridisation permits the scoring of exchange aberrations between the target chromosome and other chromosomes.

Previous work by Goodwin *et al*. demonstrated the use of

PCC and chromosome painting for studying radiation damage in hamster-human hybrids containing a single human chromosome (Goodwin et al., 1989). However, these authors used human total genomic DNA, which does not cross hybridise to hamster DNA, to visualise the single human chromosome in the hybrid. In the present paper we demonstrate the use of chromosome painting with specific full length human chromosome probes to PCC's derived from diploid human cells. We show that this methodology can be applied to detect and to score both radiation-induced chromosome breaks and exchanges.

### Materials and methods

### Cell lines

The normal human fibroblast cell line AG1522 was obtained from Dr Michael Cornforth (Los Alamos National Laboratory, Los Alamos, NM 87545). Human tumour cell lines, HeLa and HT1080, were obtained from Dr Joel Bedford (Colorado State University, CO 80523) and the American Culture Collection, respectively. AG1522 cells were maintained at low passage in α-MEM (GIBCO, Grand Island, NY), supplemented with 15% foetal bovine serum (FBS). HeLa and HT1080 cells were grown in the same medium supplemented with 10% FBS.

Mitotic HeLa and HT1080 cells were partially synchronised with 2 mM hydroxyurea (HU, Sigma, St. Louis, MO) for 12 h (Cornforth & Bedford, 1983a). HU was washed from the medium, and cells were allowed to progress through the cell cycle for 7 h. Cells were arrested in mitosis with 0.1 mg ml$^{-1}$ colcemid (GIBCO) for 5 h and collected by mitotic shake off.

### Induction of premature chromosome condensation and irradiation

The protocol for cell fusion and premature chromosome condensation followed methods previously described by Cornforth (Cornforth & Bedford, 1983a) and Hittelman (Hittelman, 1981). Briefly, mitotic HeLa or HT1080 cells and AG1522 cells were washed several times in phosphate buffered saline with 0.2 mg ml$^{-1}$ colcemid, 1 mM $CaCl_2$ at pH 7.3. Mitotic cells were mixed with interphase cells in a small volume at approximately a 1:1 to 2:1 ratio. Cells were incubated with 160 haemagglutinating units Sendai virus (kindly supplied by Dr Joel Bedford, Colorado State University) at 4°C for 15 min and then at 37°C for 10 min. Growth media (α-MEM, 20% FBS, 0.2 mg ml$^{-1}$ colcemid, 1 mM $Mg^{++}$, 10 mM 1,4 piperazinediethanesulfonic acid (PIPES), pH 6.8) was added, the pH was lowered to 6.8, and the cells were incubated for another 45 min. The cells were centrifuged and chromosome spreads were prepared by standard procedures. Cells were swollen with 0.2% KCl, 0.2% Na citrate, and 10% FBS and then fixed in methanol/acetic acid (3:1, vol/vol) and dropped onto cleaned microscope slides. Slides were stored at −20°C under nitrogen to preserve hybridisation efficiency. Before hybridisation, slides were baked at 65°C for 4 h.

For initial radiation damage studies density inhibited AG1522 cells were trypsinised, incubated with virus at 4°C, and then γ-irradiated with $^{137}Cs$ γ-rays at a dose rate of 2 Gy min$^{-1}$ before PCC induction. For repair studies attached cells were irradiated and allowed to repair at 37°C for the desired time before trypsinisation and PCC induction.

### Fluorescence *in situ* hybridisation

Human chromosome libraries were obtained from Lawrence Livermore National Laboratory, Livermore, CA. Chromosome probes were prepared by nick translation with biotin-dATP (Bethesda Research Laboratories, Gaithesburg, MD).

*In situ* hybridisation was accomplished using a modification of the procedure described by Pinkel et al. (1988). Slides containing PCC spreads were denatured in 70% formamide in 2 × SSC (0.3 M NaCl and 0.03 M Na citrate), pH 7.0 for 2 min at 70°C and dehydrated in sequential incubations of 70%/85%/100% ethanol at room temperature. A 10 µl hybridisation mixture consisting of 50% formamide, 10% dextran sulfate, 2 × SSC, 500 ng carrier DNA (sheared salmon testes, Sigma, St. Louis, MO), and 50–100 ng biotin-labelled probe, was prepared for each 22 mm$^2$ coverslip. Different amounts of unlabelled genomic DNA, based on the probe concentration and the fraction of the genome the probe represents (Pinkel et al., 1988), were added to the hybridisation mix to deplete the labelled copies of shared sequences and thereby increase contrast. The hybridisation mix was incubated for 5 min at 70°C and prehybridised for 1–2 h at 37°C before being applied to slides under a glass coverslip. After hybridisation for 3–7 days at 37°C, slides were washed in three changes of 50% formamide in 2 × SSC, pH 7.0 at 45°C, once in 2 × SSC, and once in PN buffer (0.1 M $NaH_2PO_4$, 0.1 M $Na_2HPO_4$, pH 8.0 with 0.1% Nonidet P-40). Staining was accomplished by applying alternating layers of avidin-fluorescein and biotinylated goat anti-avidin (Vector Labs, Burlingame, CA), each at 5 µg ml$^{-1}$ in PMN buffer (PN buffer, 5% non-fat dry milk). Between avidin and goat anti-avidin treatments, slides were washed 2 min in three changes of PN buffer. A fluorescein anti-fade solution (Johnson & de C. Nogueira Araujo, 1981) containing 1 µg ml$^{-1}$ propidium iodide was applied (2 µl cm$^{-2}$ under a no. 1 coverslip). The slides were viewed with a Nikon Optiphot fluorescent microscope equipped with a Nikon UFX-IIA camera system.

### Results

Low passage AG1522 cells, a normal diploid human fibroblast which exhibits density dependent inhibition, were used for all the *in vitro* studies. These cells not only provide a relatively pure population of $G_1$ cells for studies of initial chromosome damage, but also allow repair studies to be done without cells progressing through the cell cycle. Sufficient numbers of mitotic inducer cells were obtained by partially synchronising cells before collection by mitotic shake off. UV inactivated Sendai virus was used as a fusogen at concentrations which gave primarily two and three cell fusions. Since CHO cells do not cross hybridise to human probes, we performed the initial experiments with CHO mitotic cells in order that only human PCC's would be visualised. However, since diploid CHO cells were poor inducers of PCC's for AG1522 cells, we have elected to use human cells as mitotic inducers in our studies. Both HeLa cells and HT1080 cells, a diploid human fibrosarcoma cell line, gave a high efficiency of fusion and high quality PCC's. Figure 1 shows a Giemsa stained PCC between a HT1080 mitotic cell and an AG1522 $G_1$ cell, and although the individual chromosomes are distinguishable from one another, it would be difficult to precisely quantify the number of pieces as well as identify a specific chromosome of interest.

Since the technique for hybridising metaphase chromosomes with specific probes has been established (Pinkel et al., 1988), our initial approach was to apply this protocol directly to PCC slides. Since the chromosome libraries contain both unique and repetitive sequences, which are shared by all the other chromsomes, successful hybridisations to selectively 'paint' a single chromosome requires prehybridisation with unlabelled probe to block those repetitive sequences, as well as having both sufficient probe concentration and time for hybridisation. Successful metaphase chromosome hybridisation can be achieved with 1 ng µl$^{-1}$ of probe, a ratio of unlabelled to labelled probe (defined as Q) of 2, a prehybridisation time of 1–2 h, and hybridisation time of 4–5 days. Short term hybridisation (18–24 h) requires a probe concentration of 13.5 ng µl$^{-1}$ with Q = 5 (Pinkel et al., 1988). Hybridisation is visualised by staining the biotinylated probe with avidin-fluorescein and then amplifying the signal with biotinylated anti-avidin followed by another round of avidin-

**Figure 1** PCC's from a $G_1$ cell appear as single, elongated chromosomes. Conventional Giemsa staining. × 800.

**Figure 2** PCC and metaphase chromosomes hybridised with a human chromosome 4 library (Probe #4) and stained with FITC. × 5400.

**Figure 3** Hela induced PCC in an unirradiated cell hybridised with Probe #4. Note a nonreciprocal chromosome 4 rearrangement in the HeLa mitotic cell. × 680.

**Figure 4** Single unrejoined break in one chromosome 4 from a cell irradiated with 5 Gy with 24 h of repair. × 800.

**Figure 5** Two breaks, one in each chromosome 4, in cells irradiated with 2.5 Gy. × 950.

**Figure 6** Translocation between chromosome 4 and another chromosome in a cell irradiated with 2.5 Gy with 30 min of repair. × 950.

flourescein. Biotinylated probe is therefore seen as yellow-green while the rest of the DNA is counterstained with propidium iodide and seen as orange-red. However, when either of these conditions were used for PCC's, the PCC's were weakly stained and had lower signal intensities and poorer contrast compared to metaphase chromosomes. Since the HT1080 and HeLa inducer cells are also human, the hybridised metaphase chromosomes could be directly compared to the PCC's and served as an internal control for the hybridisation reaction itself. We therefore increased the probe concentration first to 2 ng $\mu l^{-1}$, then to 5 ng $\mu l^{-1}$, and finally to 10 ng $\mu l^{-1}$ for 5 days with $Q = 2$. Successful short term hybridisations (3 days) were achieved using probe concentrations of 15 ng $\mu l^{-1}$ and $Q = 3$.

Figure 2 shows a high magnification photomicrograph of a portion of a PCC spread between a mitotic HT1080 cell and a $G_1$ AG1522 cell hybridised with a human chromosome 4 specific library. It can be seen that hybridisation with this library completely covers both arms of the chromosome with only the centromeric region remaining unstained. Figure 3 shows a lower magnification photograph of an unirradiated AG1522 cell fused with a HeLa mitotic cell and hybridised with chromosome 4 probes. This HeLa cell line carries a stable nonreciprocal translocation on chromosome 4 which is readily seen.

The detection of radiation induced chromosome breaks is demonstrated in Figures 4–6. A single, unrejoined break in chromosome 4 from an AG1522 cell irradiated with 5 Gy and allowed to repair for 24 h is easily visualized in Figure 4. Figure 5 shows two breaks, one in each chromosome 4, from a cell irradiated with 2.5 Gy without any repair time.

Chromosome painting with PCC's readily lends itself for the detection of exchange aberrations which arise following chromosome rejoining. Exchange aberrations are seen as 'hybrid' chromosomes, which are stained both with fluorescein (yellow-green) and with propidium iodide (red). Figure 6 depicts an exchange aberration between chromosome 4 and another chromosome after the AG1522 cells were irradiated with 2.5 Gy and allowed to repair for 30 min at 37°C.

### Discussion

Fluorescence *in situ* hybridisation (FISH) with specific full length chromosome probes ('chromosome painting') is a powerful technique with many potential applications to cytogenetics. We have combined FISH with the premature chromosome condensation technique in order to solve the problems associated with scoring ionising radiation damage in PCC's. We have shown that the technique works well with slight modifications of the protocol already used for hybridising to metaphase chromosomes and enables breaks and translocations to be readily detected in individual prematurely condensed chromosomes. Increasing the probe concentration from the 1–2 ng $\mu l^{-1}$ used for metaphase chromosomes to 10 ng $\mu l^{-1}$ and hybridising for a minimum of 5 days resulted in readily distinguishable PCC's which are uniformly labelled. A chromosome 4 specific library was used for our initial studies, and we are currently screening all the remaining available libraries for their potential use in PCC analysis.

In regards to scoring radiation damage, this study extends the earlier work of Goodwin *et al.* (1989) who scored damage in a hamster-human cell line with a single human chromosome by hybridisation with labelled human genomic DNA. Chromosome breaks and exchanges are readily apparent and easily scored in human diploid cells with chromosome specific probes. Even spreads of inferior quality, in which not all the chromosomes are separated, can be scored because of the selective staining of a specific chromosome. It is obvious, however, that some types of damage, such as inversions or small interstitial deletions may not be detected by this technique. However, exchange aberrations, which are frequently not scored during PCC analysis because of both the difficulty associated with banding and the poor definition of the centromeric region, can easily be seen with this technique.

By using a single chromosome library of probes and therefore focusing on a particular chromosome, the effective target size for ionising radiation is decreased by the ratio of the DNA in that chromosome to the total DNA. Hence, in order to achieve results comparable to those from experiments scoring all the chromosomes, the number of PCC's scored will need to be increased by the same ratio. This is not a problem since the speed and ease of scoring the 'painted' PCC's is increased as compared to the standard method of counting the total number of fragments by a factor considerably in excess of the above ratio. However, a way to increase the target size is to combine probes so that several chromosomes are stained each with a different fluorochrome. Lucas (personal communication) has determined that multiple probes can be mixed together to increase the efficiency of scoring translocations in metaphase preparations.

The decrease in target size becomes an advantage when higher radiation doses are used (Goodwin *et al.*, 1989), such as would be needed to overlap the dose range in which DNA double strand breaks are measured by neutral elution or pulse-field gel electrophoresis (PFGE). For example, Cornforth (Cornforth & Bedford, 1983*b*) determined that 0.059 breaks/cell were produced per cGy in Ag1522 cells. Therefore, at a dose of 20 Gy, at which DNA double strand breaks are easily measured by neutral elution or by PFGE, approximately 120 breaks would be produced (which would give rise to a total of over 160 chromosome pieces). If PCC's from cells irradiated with 20 Gy were hybridised to a library specific for chromosome 4, which represents 0.067 of the genome, then only eight breaks would be produced in this chromosome (giving rise to a manageable ten fluorescent pieces to score per cell).

Cornforth and Bedford (Cornforth & Bedford, 1983*a*) introduced the technique of differential staining of the inducer and target chromosomes so as to avoid the potential artifact of a metaphase inducer cell hybridising with a contaminating interphase inducer cell. This need for differential staining still exists with FISH if human cells are used as both the inducer and target cells. The incorporation of bromodeoxyuridine (BU) into the inducer population is used to distinguish cells because BU can affect subsequent Giemsa staining. BU differential staining can also be used with fluorescently stained chromosomes since the fluorescence of the DNA dye Hoechst 33258 is quenched when BU is incorporated. Chromosomes grown in the presence of BU appear dull compared to the chromosomes without BU when stained with Hoechst 33258 and viewed under uv light. However, a more elegant solution is possible if nonhuman mitotic cells can be used as the inducer population, as the fluorescent probes do not hybridise to nonhuman cells. Although CHO cells do not give efficient PCC induction, we have recently fused CHO cells to themselves and selected a tetraploid population by flow cytometry. Using these tetraploid CHO cells as mitotic inducers has resulted in efficient PCC induction in AG1522 fibroblasts.

For many solid tumours, the treatment of choice is local radiotherapy. At present, there is no reliable method of predicting which tumours are sensitive and which are resistant. However, several recent studies are attempting to do this based on the intrinsic radiosensitivity of the tumour cells *in vitro* (Brock *et al.*, 1990; Schwartz *et al.*, 1990). Scoring chromosome aberrations *in situ* from a biopsy taken after the first treatment would provide an alternative approach, combining both intrinsic radiosensitivity and environmental factors that affect survival to radiation (such as hypoxia). PCC analysis offers distinct advantages over conventional analysis for this approach, but it requires the successful fusion between tumour cells and mitotic inducer cells, which often requires optimising the fusion conditions for each tumour cell sample. The isolation and purification of condensation factors for both DNA and chromosomes would eliminate many of the technical difficulties associated with PCC's. The problem of analysis is also complicated by tumours being both aneuploid and frequently displaying a high degree of rearrangement before treatment regimens. Chromosome painting of a nonrearranged and diploid chromosome would clearly improve the PCC analysis and may make the development of such a technique as a predictive assay of tumour radiosensitivity a feasible approach. An appropriate chromosome probe could be selected by analysing fluorescent *in situ* hybridisations of different chromosome libraries to cells from a pretreatment biopsy specimen.

With the successful development of this technique, we are now in the process of determining the initial radiation dose response of contact inhibited AG1522 cells with specific chromosome probes and comparing the yields with those determined by conventional PCC analysis using differential Giemsa staining. In addition, we are particularly interested in the repair of chromosome breaks and the rate of formation of exchange aberrations. Also, we will be able to investigate a recent report based on banding analysis of metaphase chromosomes from gamma irradiated lymphocytes which suggests that there is a nonrandom distribution of exchange sites (Barrios *et al.*, 1989). Chromosomes 1, 3, and 7, show higher than expected frequencies while chromosomes 13, 15, 21, 22, and Y show lower than expected frequencies. By combining the PCC technique with chromosome painting, it will be possible to determine if the initial damage is produced randomly in chromosomes, as expected, and whether there is a nonrandom exchange between chromosomes during rejoining.

We thank Joel Bedford for supplying Sendai virus and Rick Segraves for help with the fluorescent *in situ* hybridisation technique. We gratefully acknowledge Jeff Adams for his technical assistance and Cherry Adachi for the preparation of this manuscript. This investigation was supported by grant CA15201 from the US National Cancer Institute, DHHS.

### References

AULA, P. (1973). Virus-induced premature chromosome condensation (PCC) in single cells and G-bands of PCC-chromatin. *Hereditas*, **74**, 81.

BARRIOS, L., MIRO, R., CABALLIN, M. & 4 others (1989). Cytogenetic effects of radiotherapy. *Cancer Genet. Cytogenet.*, **41**, 61.

BEDFORD, J.S., MITCHELL, J.B., GRIGGS, H.G. & BENDER, M.A. (1978). Radiation-induced cellular reproductive death and chromosome aberrations. *Radiat. Res.*, **76**, 573.

BROCK, W.A., BAKER, F.L., WIKE, J.L., SIVON, S.L. & PETERS, L.J. (1990). Cellular radiosensitivity of primary head and neck squamous cell carcinomas and local tumor control. *Int. J. Radiat. Oncol. Biol. Phys.*, **18**, 1283.

CARRANO, A.V. (1973). Chromosome aberrations and radiation-induced cell death II. Predicted and observed cell survival. *Mutat. Res.*, **17**, 355.

CORNFORTH, M.N. & BEDFORD, J.S. (1983*a*). High resolution measurement of breaks in prematurely condensed chromosomes by differential staining. *Chromosoma*, **88**, 315.

CORNFORTH, M.N. & BEDFORD, J.S. (1983*b*). X-ray-induced breakage and rejoining of human interphase chromosomes. *Science*, **222**, 1141.

GOODWIN, E., BLAKELY, E., IVERY, G. & TOBIAS, C. (1989). Repair and misrepair of heavy-ion-induced chromosomal damage. *Adv. Space Res.*, **9**, 83.

HITTELMAN, W. (1981). Premature chromosome condensation for the detection of mutagenic activity. In *Cytogenetic Assays of Environmental Mutagens*, Hsu, T.C. (ed.) p. 353. Allanheld: Totowa, NJ.

HITTELMAN, W.N., PETKOVIC, I. & AGBOR, P. (1988). Improvements in the premature chromosome condensation technique for cytogenetic analysis. *Cancer Genet. Cytogenet.*, **30**, 301.

JOHNSON, G.D., de C. NOGUEIRA ARAUJO, G.M. (1981). A simple method of reducing the fading of immunofluorescence during microscopy. *J. Immunol. Methods*, **43**, 349.

JOHNSON, R.T. & RAO, P.N. (1970). Mammalian cell fusion: induction of premature chromosome condensation in interphase nuclei. *Nature*, **226**, 717.

LUCAS, J.N., TENJIN, T., STRAUME, T. & 4 others (1989). Rapid human chromosome aberration analysis using fluorescence *in situ* hybridisation. *Int. J. Radiat. Biol. Relat. Stud. Phys. Chem. Med.*, **56**, 35.

NATARAJAN, A.T. & OBE, G. (1978). Molecular mechanisms involved in the production of chromosomal aberrations. *Mutat. Res.*, **52**, 137.

PINKEL, D., LANDEGENT, J., COLLINS, C. & 4 others (1988). Fluorescence *in situ* hybridisation with chromosome-specific libraries: detection of trisomy 21 and translocations of chromosome 4. *Proc. Natl Acad. Sci. USA*, **85**, 9138.

PINKEL, D., STRAUME, T. & GRAY, J. (1986). Cytogenetic analysis using quantitative, high sensitivity, fluorescence hybridisation. *Proc. Natl Acad. Sci. USA*, **83**, 2934.

REVELL, S.H. (1983). Relationship between chromosome damage and cell death. In *Radiation-Induced Chromosome Damage in Man*. Ishihara, T. & Sasaki, M. (eds) p. 215. Liss: New York.

SCHWARTZ, J.L., MUSTAFI, R., BECKETT, M.A. & WEICHSELBAUM, R.R. (1990). Prediction of the radiosensitivity of human squamous cell carcinoma using DNA filter elution. *Radiat. Res.*, **123**, 1.

SPERLING, K. & RAO, P.N. (1974). Mammalian cell fusion V. Replication behaviour of heterochromatin as observed by premature chromosome condensation. *Chromosoma*, **45**, 121.

WLODEK, D. & HITTELMAN, W.N. (1988). The relationship of DNA and chromosome damage to survival of synchronized x-irradiated L5178Y cells. *Radiat. Res.*, **115**, 550.